# Electroencephalogram approximate entropy influenced by both age and sleep

**DOI:** 10.3389/fninf.2013.00033

**Published:** 2013-12-05

**Authors:** Gerick M. H. Lee, Sara Fattinger, Anne-Laure Mouthon, Quentin Noirhomme, Reto Huber

**Affiliations:** ^1^Institute of Neuroinformatics, University of Zurich and ETH ZurichZurich, Switzerland; ^2^Child Development Center, University Children's Hospital ZurichZurich, Switzerland; ^3^Coma Science Group, Neurology Department, Cyclotron Research Centre, University Hospital of Liège, University of LiègeLiège, Belgium

**Keywords:** electroencephalogram, development, sleep, consciousness, approximate entropy

## Abstract

The use of information-based measures to assess changes in conscious state is an increasingly popular topic. Though recent results have seemed to justify the merits of such methods, little has been done to investigate the applicability of such measures to children. For our work, we used the approximate entropy (ApEn), a measure previously shown to correlate with changes in conscious state when applied to the electroencephalogram (EEG), and sought to confirm whether previously reported trends in adult ApEn values across wake and sleep were present in children. Besides validating the prior findings that ApEn decreases from wake to sleep (including wake, rapid eye movement (REM) sleep, and non-REM sleep) in adults, we found that previously reported ApEn decreases across vigilance states in adults were also present in children (ApEn trends for both age groups: wake > REM sleep > non-REM sleep). When comparing ApEn values between age groups, adults had significantly larger ApEn values than children during wakefulness. After the application of an 8 Hz high-pass filter to the EEG signal, ApEn values were recalculated. The number of electrodes with significant vigilance state effects dropped from all 109 electrodes with the original 1 Hz filter to 1 electrode with the 8 Hz filter. The number of electrodes with significant age effects dropped from 10 to 4. Our results support the notion that ApEn can reliably distinguish between vigilance states, with low-frequency sleep-related oscillations implicated as the driver of changes between vigilance states. We suggest that the observed differences between adult and child ApEn values during wake may reflect differences in connectivity between age groups, a factor which may be important in the use of EEG to measure consciousness.

## 1. Introduction

Recent theoretical work has proposed a link between the ability of the brain to integrate information and its corresponding conscious state (Tononi and Sporns, 2003; Tononi, 2004, 2008, 2012; Balduzzi and Tononi, 2008). Meanwhile, related experimental work has shown a link between changes in informational processing and conscious state (Massimini et al., 2005, 2007; Ferrarelli et al., 2010; Casali et al., 2013). These studies have provided compelling evidence of a causal relationship between the complexity of neural responses to external stimulation, as measured with the electroencephalogram (EEG), and the conscious state of the subject. Nevertheless, the benefits (particularly in the clinical setting) of a metric for conscious state independent of external stimulation are enough to encourage further work toward such a measure.

In this search for an EEG-specific measure of consciousness, many information-based measures have been applied. For this study, we chose the approximate entropy (ApEn), a measure of regularity in the time domain. Originally designed for use on physiological data (Pincus, 1991), ApEn quantifies the predictability of a signal by comparing the number of matching sequences of a given length with the number of matching sequences one increment (time bin) longer. It has been suggested as an EEG measure of conscious state, and ties into informational theories of consciousness. Theoretical analysis has shown that isolated systems should show decreases in ApEn values (Pincus, 1994). This concurs with findings that non-rapid eye movement (NREM) sleep, associated with decreases in consciousness (Stickgold et al., 2001), tends to feature long-distance connectivity decreases and increases in local clustering (Massimini et al., 2005, 2007; Ferri et al., 2007, 2008; Spoormaker et al., 2010; Uehara et al., 2013). Rapid eye movement (REM) sleep, a state similar to wakefulness in its content of conscious experience, tends to show functional connectivity patterns more similar to those of wake (Massimini et al., 2010).

Prior applications of ApEn as a measure of conscious state have successfully shown correlations with anesthetic depth (Rezek and Roberts, 1998; Bruhn et al., 2000a,b; Zhang et al., 2001; Bruhn et al., 2003; Li et al., 2008; Hayashi et al., 2012), though these findings were contradicted by Jordan et al. (2006), who failed to report certain key parameters. Burioka et al. (2005) applied ApEn to data from adults across wake and sleep, finding a consistent decrease in ApEn from wake to sleep, with the lowest values occurring during deep sleep. Gu et al. (2003) also applied ApEn to data across multiple stages of sleep, and during epileptic seizure onset, reporting decreases during sleep and during seizure onset, but did not use any statistical testing. Attempts to tie ApEn changes to behavioral changes during wakefulness have found conflicting results: ApEn analysis of subjects driving while sleep deprived found no significant changes in ApEn preceding driving errors (Papadelis et al., 2007a), though Flores Vega et al. (2013) recently showed that ApEn could be used to differentiate between some of the various mental tasks tested. Papadelis et al. (2007b) found no significant changes in ApEn as a function of hypoxia, but ApEn derived metrics did show significant changes. In summary, though its resolution within the wake state is unclear, when analyzing subjects between wakefulness and other conscious states, ApEn values consistently decreased with loss of consciousness. Comparisons of ApEn with other information-based measures typically showed it to be of comparable accuracy and reliability (Rezek and Roberts, 1998; Zhang et al., 2001; Bruhn et al., 2003; Abásolo et al.,^2008^; Li et al., 2008; Anier et al., 2012).

Past work has documented changes in EEG power across development, during both sleep (Feinberg, 1983; Buchmann et al., 2010; Feinberg and Campbell, 2010; Kurth et al., 2010) and wake (Whitford et al., 2007). To our knowledge, no group has yet applied ApEn to the EEG data of children. Therefore, to further assess the merits of ApEn as a measure of conscious state, we applied ApEn to EEG data recorded across sleep and wake, from both adults and children. Besides replicating the finding that ApEn can mark changes in vigilance state due to sleep in adults, we sought to verify that similar ApEn trends were present across wake and sleep in children, while also assessing any impact of age on ApEn values across both wake and sleep.

## 2. Materials and methods

### 2.1. Subjects

For this study, subjects were pooled into two age groups of six subjects each, hereafter referred to as adults (age range: 19.4–25.1 years; mean age ± SD: 23.2±2.06 years; 0 females), and children (age range: 10.6–12.6 years; mean age ± SD: 11.4±0.691 years; 2 females). Subjects wore wrist actigraphs and kept sleep diaries to ensure sleep schedule compliance. Napping, alcohol consumption, and taking medication were all forbidden for the 24 h preceding the recording. Informed written consent was obtained from all subjects or their legal guardians. All procedures were performed with approval of the local ethics committee, and in accordance with the Declaration of Helsinki.

### 2.2. Data acquisition

All data (EEG, electrooculogram, and electromyogram) were gathered previously by our group at the University Children's Hospital Zurich during one evening, night, and morning. All sleep data used were originally published in earlier studies from our group (Kurth et al., 2010, 2012). Of the data recorded previously, subjects within the selected age range and with minimally-artifacted data were used, particularly during wakefulness and REM sleep. Wake data have not yet been used for publication, and were recorded during an auditory oddball task that was performed shortly before and after full night sleep recording. Subjects were awoken at a time allowing for normal school or work attendance. A 128-electrode high-density EEG array (Electrical Geodesics, Eugene, OR, USA) was used for recording, with a sampling frequency of 500 Hz. Electrodes were referenced to the vertex during recording, which was used for filtering, downsampling, and artifact rejection. Impedences were set below 50 kΩ. Data were divided into 20 s epochs, the sleep stages of which were categorized using standard criteria (Iber et al., 2007). For the scoring of sleep stages, the recordings were referenced to the mastoid electrodes.

For analysis with the ApEn algorithm, data were then bandpass filtered at frequencies of 1 and 35 Hz, respectively, and downsampled to 250 Hz before being corrected for artifacts. Artifact correction for sleep data involved visual inspection of the power between 0.75–4 Hz, and 20–30 Hz, rejecting individual channels for a given epoch if the power exceeded a mean band power value. Artifact correction for wake data was based on independent component analysis, as presented by Jung et al. (2000). Finally, data were referenced to the average activity of all non-rejected channels above the ears for analysis. To investigate better the role of low-frequency EEG activity on ApEn, we later refiltered our original data with an 8 Hz high-pass filter, and recalculated the ApEn.

ApEn analysis used all 109 electrodes above the ears not rejected during artifact correction. Data preprocessing and all analyses were done using Matlab (The MathWorks, Natick, MA, USA), statistical testing used Matlab, as well as R (R Foundation for Statistical Computing, Vienna, Austria). Data series of 4000 points, corresponding to 16 s of EEG signal, were used for analysis. Because wake epochs were scored in epochs of 4 s duration, analysis used aggregate 16 s epochs comprised of four consecutive artifact-free epochs, taken from the evening recording session preceding sleep. Sleep data was drawn from the first 16 s of unartifacted 20 s epochs. Sleep epochs used were preceded and followed by at least 1 min (three epochs) of sleep all of the same stage, to minimize the influence of stage transitions. For one adult subject, only two epochs (40 s) preceded and followed the epoch for the N3 sleep stage used for all analyses.

### 2.3. Approximate entropy (ApEn)

The development of ApEn was driven by the need for a distribution-free measure of signal regularity. Unlike the Shannon entropy, the calculation of ApEn is not predicated on the underlying distribution of the data; it is instead based on sequence recurrence. This allows ApEn to be applied to signals of shorter length, and makes model estimation wholly unnecessary, removing the risk for misestimation based on poor model selection.

ApEn can be understood as the logarithmic ratio of component-wise matching sequences from a signal of length *N*. The other relevant parameters are *r*, a factor based on the standard deviation of the signal being analyzed, and used for comparison. The final parameter is *m*, the length of sequences compared. It is measured as an integer count of discrete time bins. The ApEn is computed as follows:

1. The first sequence of length *m*, is compared with all other sequences of the same length in a point-wise manner. Those sequences for which all points are within *r* of their corresponding point in the original sequence are counted as a match (including the base sequence with itself). This count is used in step 3.
2. The same comparison is made for sequences of length *m* + 1, starting with the first sequence of *m* + 1 points. This count is used in step 3.
3. The count from step 2 is divided by step 1, and the natural logarithm of this ratio is taken.
4. This process is then repeated for all possible sequences (the final *m* points of the signal cannot be used, as there would be no *m* + 1 sequence for comparison).
5. All logarithm results are then summed, divided by *N* − m (the total number of possible base sequences), and multiplied by −1.

The minimum value for ApEn is 0, suggesting a fully predictable sequence. ApEn values are heavily dependent on parameter choice, and values calculated with different parameter choices cannot be compared. Because the filter factor, *r*, typically has its values pegged to the standard deviation of the sequence, the origin of ApEn's robustness to noise and scale invariance can be seen. Our parameters were set per the suggestion of Pincus and Goldberger (1994), as well as other groups applying ApEn to EEG data (Bruhn et al., 2000a; Li et al., 2008; Hayashi et al., 2012), specifically Burioka et al. (2005), to *m* and *r* values of 2 and 0.2 · SD, respectively. Our *N* value, the length of the data series used, was 4000 points.

To confirm the proper functioning of the ApEn algorithm, we computed ApEn values for six regular sine curve sequences of 4000 points, with a simulated sampling rate of 250 Hz. The sine frequencies used were 1, 2, 4, 8, 16, and 32 Hz, frequencies all within the range used in our EEG ApEn analysis. Each sine curve sequence was then randomly shuffled twenty times. ApEn values were calculated for all six sine curve sequences, and all 120 random sequences (twenty random ApEn values per sine curve).

For an excellent appendix detailing the steps in ApEn calculation (including a simple by-hand walkthrough of the steps involved in ApEn calculation, as well as a sample implementation in Visual Basic), please see Bruhn et al. (2000a).

### 2.4. Statistical analysis

As mentioned above, statistical analyses were performed using Matlab and R. Values were imported into R and log-transformed, to better approximate a normal distribution. A linear mixed model for the subject age groups (independent factor) and vigilance states (repeated-measures factor) was then generated and tested using a repeated-measures ANOVA.

All multiple comparisons corrections were performed using the Holm–Bonferroni method. Because EEG electrodes are not independent, the Holm–Bonferroni correction is overly conservative. For this reason, in order to provide the most informative results, *p*-values and significance results from comparisons using all electrodes are reported both with and without correction. To better investigate differences between age groups, unpaired independent-samples *t*-tests were performed between each age group within each vigilance state.

## 3. Results

As described above, we analyzed a set of simulated data to validate our ApEn algorithm. ApEn values for the simulated data ranged between 0.07 and 0.29 for the sine curves. Mean ApEn values for the shuffled sequences were all 1.94, with standard deviations of less than 0.01. These results were in line with expectations.

**Figure 1:**
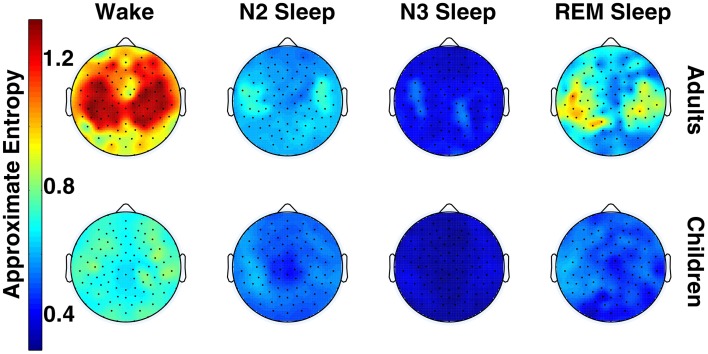
**Topography of across-subject mean ApEn values across vigilance state in adults and children (for both groups, *n*=6)**.

**Figure 2:**
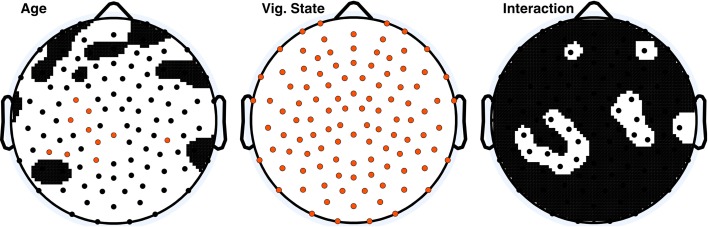
**ANOVA *p*-value distributions; electrodes with factor effect *p*-values less than 0.05 are those displayed in black on the white background**. Electrodes with significant *p*-values following Holm–Bonferroni correction are depicted in orange. The nasion electrode (not shown) was significant for the vigilance state effect *post-hoc*, and was not significant at any level for the other effects.

Figure 1 shows the topographical distribution of mean ApEn per electrode in adults and children. ApEn value trends across vigilance states were similar for both age groups, and were as follows: wake ApEn > REM sleep ApEn > N2 sleep ApEn > N3 sleep ApEn, though REM sleep and N2 sleep were often overlapping, especially in children. Figure 2 displays the ANOVA results for all factors. All 109 tested electrodes had significant vigilance state effects after *post-hoc* correction. Ninety-three electrodes, widely distributed across the scalp, showed a significant age effect before correction. Ten electrodes had a significant age effect after correction. These ten electrodes were largely clustered over the left parietal and the area between the occipital and temporal lobes, with one isolated over the right temporal lobe.

**Figure 3:**
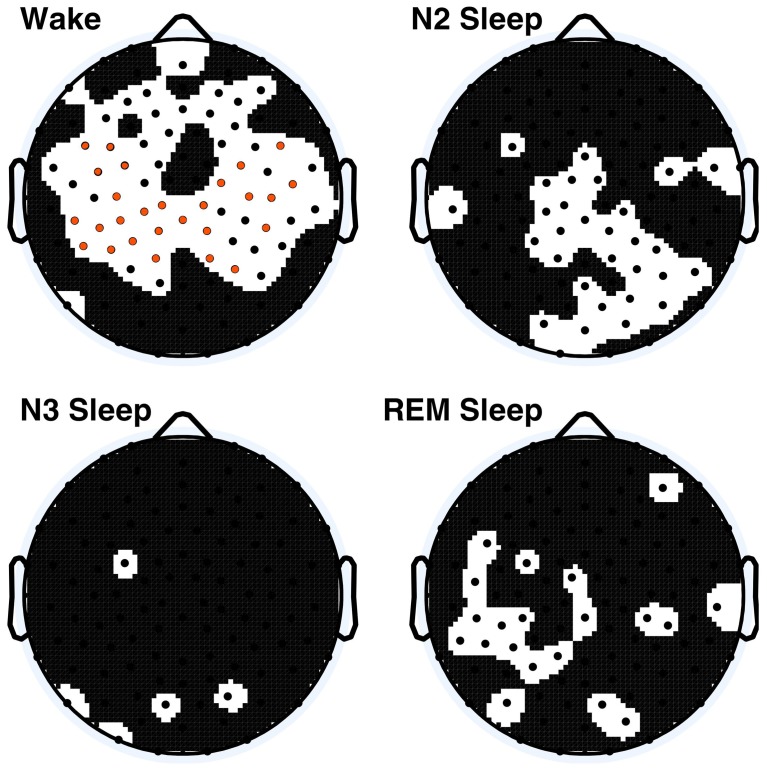
**Within vigilance state *t*-test *p*-values across all electrodes, electrodes for which *p* < 0.05 are displayed in black on the white background, values significant following Holm–Bonferroni correction are depicted in orange**. The nasion electrode (not shown) was significantly different during wakefulness after *post-hoc* correction.

To better discern the causes of the observed age effects, within-vigilance state pairwise *t*-tests were calculated across all electrodes. These results are shown in Figure 3, where 66 electrodes had significant age effects during wakefulness before correction, of which 28 electrodes were still significant following correction. N2 sleep and REM sleep had large clusters of significant electrodes before correction; none were significant after correction.

To fully explore the possibility that sleep regulatory differences between age groups may influence our results (Carskadon et al., 1980; Carskadon and Acebo, 2002), and to verify that ApEn wake values are not influenced by potential changes in overall synaptic weighting during sleep [as proposed by Tononi and Cirelli (2003)], we compared ApEn from both the evening and morning recording sessions, averaged across all 109 electrodes. A Two-Way ANOVA for age and recording session found a significant age effect (*p*< 0.001), as expected from earlier testing, but found no significant effect for the recording session, nor for the interaction of the two (*p*> 0.1 for both effects). For all other ApEn analysis, evening wakefulness was used to represent wakefulness.

**Figure 4:**
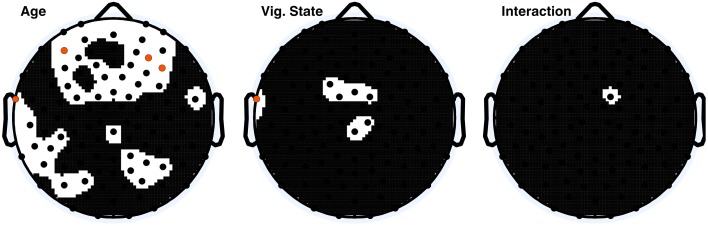
**ANOVA *p*-value distributions from 8 Hz high pass filtered data; electrodes with factor effect *p*-values less than 0.05 are those displayed in black on the white background**. Electrodes with significant *p*-values following Holm–Bonferroni correction are depicted in orange. The nasion electrode (not shown) was not significant at any level for any effect.

To assess the origin of the observed ApEn differences between children and adults, a high-pass filter of 8 Hz was applied to the data, and ApEn values were again calculated. Two-Way ANOVA results from the high-pass-filtered data of all electrodes are depicted in Figure 4. Forty-two electrodes had significant age effects before correction, of which four electrodes were significant following correction. Vigilance state effects were almost entirely abolished; seven electrodes were significant before correction; one electrode was significant after correction.

To check for changes in the regional distribution of ApEn, electrode values were normalized to the within-subject-within-vigilance-state mean across all electrodes. One electrode (located near the posterior end of the right frontal area) showed a significant vigilance state effect after correction. No other electrodes were significant for any effect (age, vigilance state, or the interaction of the two), even before *post-hoc* correction.

**Figure 5:**
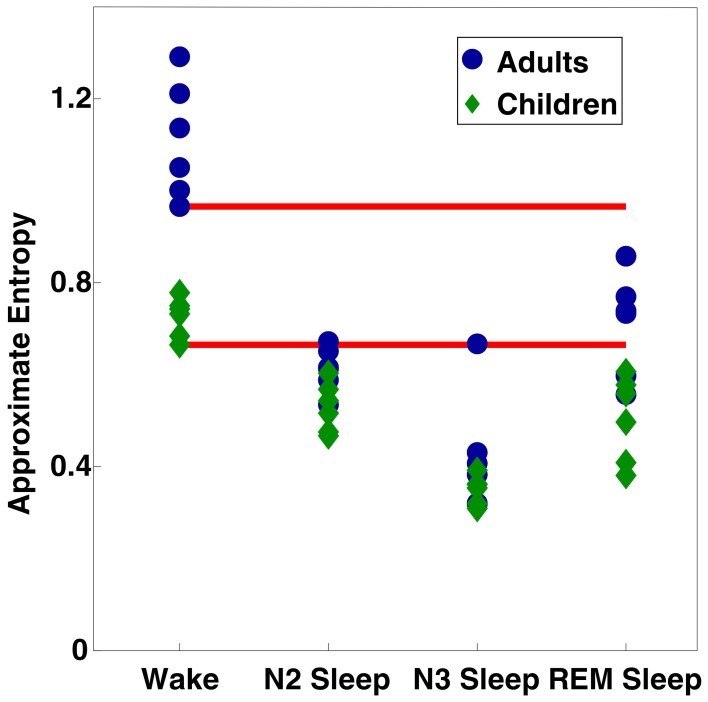
**Scatter plot of ApEn values per subject per vigilance state, averaged across all 109 electrodes**. Red bars indicate the minimum values observed for each age group during wakefulness.

Finally, to investigate individual differences in ApEn values, we averaged ApEn across all electrodes, and plotted values for each stage as Figure 5. The minimum values from wakefulness were invariably higher than the maximum observed ApEn value from sleep (including both NREM and REM sleep) within the same age group. Comparison of all subjects showed some adult sleep values (especially during REM sleep) greater than some or all wake ApEn values for children.

## 4. Discussion

Our analysis showed significant ApEn effects due both to vigilance state and age, with age differences being predominantly driven by differences during wakefulness. As a measure of vigilance state, ApEn showed strongly significant results across wake and sleep, with ApEn values in adults following the same trend as those previously reported (Burioka et al., 2005). ApEn results from children followed similar trends between vigilance states, with the only significant age differences occuring during wakefulness. As demonstrated in Figure 5, within age group minimum ApEn values for wakefulness were higher than maximum ApEn sleep values for the same age group, supporting the notion that ApEn can reliably detect changes in vigilance state. The almost complete abolition of significant vigilance state effects observed following application of the 8 Hz high pass filter to our data provide evidence that slow wave activity, the key EEG oscillation of deep (NREM) sleep (Steriade et al., 2001; Buzsaki, 2006), is also the key driver behind the increased regularity observed during sleep.

Pincus (1994) observed that isolated systems have lower ApEn values. If the brain is indeed a more segregated one during NREM sleep, as suggested by experimental work (Massimini et al., 2005, 2007), then one would expect to see decreases in ApEn during NREM sleep, as we did. These findings concur with the proposal presented in Tononi and Massimini (2008), which drew a link between slow wave activity during deep sleep and an interruption in information processing, leading to loss of consciousness. That ApEn differences due to vigilance state mostly disappeared after the removal of the lower frequency bands connects ApEn changes to the presence of sleep oscillations, specifically slow waves. Our results therefore suggest the possibility of a causal relationship between EEG signal changes, as measured via ApEn, and the hyperpolarization phase associated with the slow oscillation (Steriade et al., 2001). This hyperpolarization has been implicated in the induction of loss of consciousness (Massimini et al., 2005).

The almost complete lack of significant vigilance state differences following normalization to the mean value across all electrodes indicates that changes in ApEn values across wake and sleep are not the result of changing topographical distribution. These results were therefore unlike previously observed age-dependent topographical changes in sleep slow wave activity (Kurth et al., 2010), and rather suggest that changes in signal regularity are of a more global nature.

Besides the widely distributed nature of changes due to sleep stage, changes between wake adults and children were also found to be global: Pairwise *t*-tests found a broad distribution of electrodes with significant increases in wake ApEn values across development. These results concur with those of Gasser et al. (1988), who found absolute EEG band power decreases in the delta and theta bands (both of which were below 7.5 Hz), and the overall spectrum, across adolescence when measuring during eyes-closed wake. Our findings also agree with the EEG results of Whitford et al. (2007), who found global power decreases during wakefulness across age, especially in the lower frequency range (0.5–7.5 Hz).

While EEG power changes between adults and children have also been observed during sleep [as reviewed in Feinberg (1983); Feinberg and Campbell (2010), also Buchmann et al. (2010); Kurth et al. (2010)], we only observed age differences in ApEn values during wakefulness. This discrepancy may potentially be explained by the large increase in EEG power during sleep. EEG power differences caused by sleep-related oscillations may be of a large enough scale relative to those due to developmental changes that ApEn age differences are obscured. Figure 5, the scatter plot of individual mean ApEn values shows a tendency for ApEn values to be lower in children during sleep (the largest ApEn values for any given stage are invariably from adults; the lowest from children), even though statistical testing reveals no age differences.

Our results from wakefulness may also be in line with this claim; if ApEn age differences during wakefulness reflect anatomical connectivity changes, then the lack of significant differences at occipital and temporal electrodes is in line with what would be expected based on prior developmental research work. The review of Feinberg (1983) drew parallels between their work measuring changes in sleep EEG activity across development, and anatomical work, which showed regional variation in synaptic densities across development [Huttenlocher (1979); Huttenlocher et al. (1982), expanded in Huttenlocher and Dabholkar (1997)]. These works independently demonstrated that primary sensory cortices were first to reach adult-level values, both when measured via EEG power during sleep, and histological synaptic density counts. Coupled MRI and EEG work from our group found correlations between slow-wave activity decreases during sleep and gray matter volume decreases (Buchmann et al., 2010). Similar work during wakefulness from other groups showed correlations between gray matter volume decreases and low-frequency EEG decreases from late childhood through adulthood (subjects ranged between 10 and 30 years of age, Whitford et al., 2007), particularly in the parietal and frontal regions, where our significant differences were focused. Developmental changes in the topographical distribution of low-frequency sleep oscillations followed similar trends; regions converging to adult-level synaptic densities earlier were also the first to converge to adult-level EEG activity (Kurth et al., 2010). Without the use of other tools, such as single-unit recording or transcranial magnetic stimulation, it is difficult to separate EEG slow wave activity from the changes in functional connectivity observed on the neuronal level during slow wave sleep. Nevertheless, the decrease in ApEn observed between wakefulness in children and in adults matches with the increased local anatomical connectivity observed in children. That changes in both vigilance state and sleep result in decreased ApEn values supports the notion that changes in ApEn values may reflect connectivity changes, both anatomical and functional.

Though this claim must be further tested, if true, it would mean that ApEn changes reflect both functional (between wake and sleep) and anatomical (across development) connectivity changes in the brain. As we have shown, ApEn can reliably distinguish between wake and sleep within subject age groups. However, having demonstrated that age has an uneven influence on ApEn values across changes in vigilance state, we highlight the need for future research to fully explore the influence of age on proposed information-based EEG measures of consciousness.

## Author contributions

Gerick M. H. Lee, Anne-Laure Mouthon, Quentin Noirhomme, and Reto Huber designed research; Gerick M. H. Lee and Sara Fattinger performed research; Gerick M. H. Lee, Sara Fattinger, and Reto Huber analyzed data; and Gerick M. H. Lee and Reto Huber wrote the paper.

### Conflict of interest statement

The authors declare that the research was conducted in the absence of any commercial or financial relationships that could be construed as a potential conflict of interest.
